# Neuromuscular and acute symptoms responses to progressive elastic resistance exercise in patients with chronic obstructive pulmonary disease: Cross-sectional study

**DOI:** 10.3389/fmed.2022.934410

**Published:** 2022-10-26

**Authors:** Joaquin Calatayud, Rodrigo Torres-Castro, Roberto Vera-Uribe, Álvaro Olivares-Valenzuela, Benjamín Guzmán-González, María E. Torres, Nicolás Sepúlveda-Cáceres, Lars L. Andersen, Carlos Cruz-Montecinos

**Affiliations:** ^1^Exercise Intervention for Health Research Group (EXINH-RG), Department of Physiotherapy, University of Valencia, Valencia, Spain; ^2^National Research Centre for the Working Environment, Copenhagen, Denmark; ^3^Department of Physical Therapy, Faculty of Medicine, University of Chile, Santiago, Chile; ^4^International Physiotherapy Research Network (PhysioEvidence), Barcelona, Spain; ^5^Division of Research, Development and Innovation in Kinesiology, Kinesiology Unit, San José Hospital, Santiago, Chile; ^6^Respiratory Unit, San José Hospital, Santiago, Chile; ^7^Department of Health Science and Technology, Aalborg University, Aalborg, Denmark

**Keywords:** strength training, elastic bands, COPD, neuromuscular, muscle activity, EMG

## Abstract

**Background:**

Quadriceps muscle training is a key part in the rehabilitation of chronic obstructive pulmonary disease (COPD) patients. However, exercise intensity prescription and progression with the typically used elastic bands is challenging. We aimed to evaluate neuromuscular, acute symptoms and cardiorespiratory responses (heart rate and dyspnea) during progressive elastic resistance exercise in patients with COPD.

**Methods:**

Fourteen patients diagnosed with moderate-very severe COPD performed knee extensions at different elastic resistance levels (i.e., colors). The neuromuscular activity was recorded using surface electromyography for the rectus femoris, vastus lateralis and vastus medialis, together with rate of perceived exertion, perceived quadriceps fatigue, dyspnea, oxygen saturation and heart rate.

**Results:**

For the vastus lateralis and rectus femoris, increase of muscle activity was evident from a two-level increment when using the red color. For the vastus medialis, there were no muscle activity progressions. Dyspnea, quadriceps fatigue and especially rate of perceived exertion increased in a dose-response fashion and were correlated with the resistance level and muscle activity at the three muscles.

**Conclusion:**

Heavy elastic resistance exercise is feasible in COPD patients without excessive dyspnea and a stable cardiorespiratory response. In general, at least two elastic resistance increments are needed to enhance muscle activity for the vastus lateralis and rectus femoris, while there is no increase for the vastus medialis. These results may help to individualize exercise dosing during elastic resistance training in patients with COPD.

## Introduction

Chronic obstructive pulmonary disease (COPD) is a common, preventable, and treatable disease that is characterized by airflow limitation and persistent respiratory symptoms caused by significant cigarette smoke or noxious particles exposure (1). It has been estimated that COPD will be one of the top causes of death worldwide in 2030 (2, 3).

The main symptom of COPD is chronic and progressive dyspnea (4). Still, several systemic manifestations as cardiovascular disease or limb muscle dysfunction have been reported to produce a marked deterioration in patients’ quality of life alongside increased mortality (5). Limb muscle dysfunction is characterized by a decreased proportion of type I muscle fibers and oxidative capacity and reduced cross-sectional muscle area, strength, and endurance (5). Peripheral muscle weakness, particularly of the large quadriceps muscles due to their vital role in daily living activities, is an essential target of comprehensive disease management (6). The weakness of quadriceps is prevalent in approximately 50% of patients with severe/very severe COPD (7), and is associated with low exercise tolerance (8), reduced quality of life (9), increased use of health resources (10), and higher mortality risk (11). In consequence, quadriceps muscle training needs to be included in every well-designed rehabilitation program.

Quadriceps muscle training has been typically performed with the patients’ own body weight as a load or with traditional weight machines. In addition, elastic resistance is another portable, cheap and safe alternative. Importantly, the few experimental studies using elastic resistance to train the quadriceps among COPD patients reported effectiveness in improving functional capacity and muscular function (12–14). However, exercise intensity prescription and progression with the elastic bands differs between studies, likely due to a more challenging objective load quantification. Typically, changing one color to another increases resistance from 20 to 30% (15). However, there are no previous studies corroborating the number of resistance increments needed during a typical COPD rehabilitation exercise to obtain a real muscle activity increase. Furthermore, achieving sufficient neuromuscular stimulus can be difficult among COPD patients since ventilatory limitation, dyspnea (16), or muscle fatigue (17) can occur before.

The purpose of the study was to evaluate neuromuscular and acute symptoms and cardiorespiratory responses (heart rate and dyspnea) to progressive elastic resistance exercise in patients with chronic obstructive pulmonary disease. As a secondary objective, we evaluated the correlation between these variables. We hypothesized that different elastic resistance progressions would be needed to increase quadriceps muscle activity and that acute symptoms but not cardiorespiratory responses would increase.

## Materials and methods

### Participants

During November 2018 to May 2019, candidates who met the following conditions were selected for the present study: patients over 40 years of age diagnosed with stage II-IV (moderate-very severe) COPD, according to GOLD criteria (1), who were stable (i.e., no exacerbations or medication changes within 4 weeks before the study) at a local hospital (San José, Santiago Chile). The exclusion criteria included the presence of musculoskeletal, rheumatic, cardiac, or neurological disorders that might affect exercise performance, previous lung or cardiac surgery, long-term oxygen treatment, and participation in a resistance training program over two times/week within 6 months before starting the study.

All the participants were informed about the objective of the study, and we obtained their respective informed consent. This study was approved by a local ethics committee and conducted in agreement with the Declaration of Helsinki. This article adheres to the STROBE guidelines.

### Procedures

The following clinical variables and symptoms were collected from patients’ medical histories: sex, age, anthropometric characteristics (weight, height, and body mass index [BMI]), GOLD classification (1), forced expiratory volume during the first second, absolute value and percentage of predicted value based on global lung initiative (GLI) reference values (18), dyspnea measured through the modified medical research council (19), the functional exercise capacity measured through the six-minute walk distance (20), the quality of life measured with the COPD assessment test (CAT) (21) and Saint George respiratory questionnaire (SGRQ) (22), and the comorbidities measured by the Charlson index (23).

Each participant carried out one experimental session. The participants were not allowed to eat, drink or take stimulants (such as caffeine) 2 h before the session and were not allowed to perform physical activity more intense than daily life activities 24 h before the measurement. They were also recommended to sleep a minimum of 7–8 h the night before the assessments. All measurements were made by the same two researchers and were conducted in the same facility at the hospital.

The surface electromyography (sEMG) protocol started with the preparation of participants’ skin, followed by electrode placement, maximum voluntary isometric contractions (MVIC), and the performance of the different exercise conditions. Hair was removed from the skin overlying the muscles of interest, and the skin was then cleaned by rubbing with cotton wool dipped in alcohol for the subsequent electrode placement. Electrodes were placed on the rectus femoris (RF), vastus lateralis (VL) and vastus medialis (VM) muscles on the dominant leg following the SENIAM (Surface EMG for Non-Invasive Assessment of Muscles) recommendations.^1^ The dominant side was determined by the question: “Which leg do you prefer to hit a ball with?” Specifically, electrodes for the RF were fixed at 50% of the distance between the anterior superior iliac spine and the superior part of the patella in the same direction of that line. For the VM, the electrodes were placed at 80% of the line between the anterior superior iliac spine and the joint space in front of the anterior border of the medial ligament of the knee, with a perpendicular orientation to that line. For the VL, the electrodes were located at two-thirds of the line that connects the anterior superior iliac spine with the lateral side of the patella following the orientation of the muscle fibers. Bipolar electrodes (Kendall Medi-Trace Mini ECG Electrodes, Neurotronics, Randwick, NSW, Australia) were placed with an inter-electrode distance of 2 cm. One reference electrode was positioned a finger-length from the other electrodes for the RF muscle and above the patella for the VL and VM muscles, according to the manufacturer’s specifications.

Using commercial hardware and software, sEMG was recorded (MyoSystem DTS, Noraxon USA, Inc., Scottsdale, CA, USA), with a sample rate of 1,500 Hz. To normalize sEMG amplitude, the maximal voluntary isometric contraction was assessed before starting with the exercise performance. Patients were asked to complete two MVICs with a 1 min rest between trials. They performed a 2 s progressive contraction and then maintained a maximum contraction for the next 3 s. Verbal encouragement was provided to motivate all participants to reach their maximal effort. The position during the evaluation of the MVICs was based on standardized muscle testing procedures and performed against manual resistance (24). Specifically, the knee extension was performed with the participant seated with 90° knee flexion and approximately 110° hip flexion. Besides, patients maintained neutral dorsiflexion of the ankle at 90°.

**FIGURE 1:**
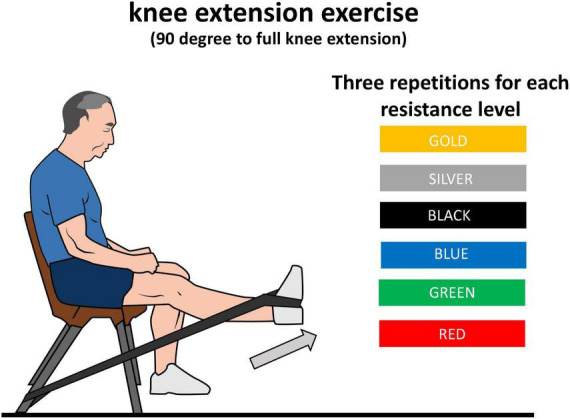
Schematic representation of knee extension exercise using different elastic resistance band colors.

After a 2 min break, the knee extension exercise was performed in the same place and in the same position above described, with six different elastic resistance levels (red, green, blue, black, silver and gold; Thera-Band CLX; The Hygenic Corp., Akron, OH, USA), performed at this order and separated by 2 min of rest. The elastic bands were fastened at the chair (Figure 1). According to the elastic band manufacturer, there is a 20% resistance difference between the different bands, except between the two hardest levels (silver and gold), where there is a 30% resistance difference. Specifically, the different resistances according to the elastic band manufacturer at 100% elongation are 1.67, 2.09, 2.63, 3.31, 4.63, and 6.44 kg for the red, green, blue, black, silver, and gold bands, respectively. The band length was 1.9 m, and they were pre-stretched to 50% of their size before performing the exercise to achieve an appropriate intensity.

The exercise was performed with the participant’s available range of motion. Participants were asked to perform three repetitions for each elastic resistance level and to move their body and trunk as little as possible and to perform the exercise smoothly without stops or accelerations. For this purpose, a metronome was used with a speed of 1.5 s for concentric contraction and 1.5 s for eccentric contraction, with a beep sound at the change of each phase. If they were not able to perform the exercise with the correct technique, the attempt was canceled and repeated. If the patient was not able to reach three repetitions during a certain condition, the experimental session finished. After performing each condition, a researcher asked each patient to rate their perceived exertion (RPE), dyspnea and perceived quadriceps fatigue (quadriceps fatigue) using the Borg CR10 scale (25). Immediately after the exercise, oxygen saturation and heart rate were measured with a pulse oximeter (Mindray, model Umec 10, Shenzhen, China). Further, the pulse oximeter was active during exercise so oxygen saturation and heart rate were constantly monitored for safety reasons.

### Surface electromyography processing

Surface electromyography (sEMG) data processing was performed using custom-made algorithms implemented in MATLAB (version R2015a; The MathWorks, Inc., Natick, MA, USA) software. For the analysis, all raw sEMG signals, obtained during the exercises, were digitally filtered with high-pass filtering at 10 Hz and a moving root-mean-square (RMS). The RMS routine was performed using a smoothing filter/window of 500 milliseconds (250 milliseconds backward and 250 milliseconds forward from each data point) across the entire signal (i.e., across all contractions) (26). In each of the muscles and for each level of exercise intensity, an RMS peak from each of the contractions was obtained (i.e., a total of three RMS peaks). These three RMS peaks were averaged, and the value obtained was then normalized to the maximum activation value reached at the MVICs, obtaining the normalized RMS (nRMS).

### Statistical analysis

Simple correlations analyses were performed at calculation Pearson’s r (correlation coefficient). The association between the resistance level and each of the physiological outcome variables were modeled using general linear models (Proc GLM, SAS). Results from these analyses were reported as the least square means at each intensity and as differences of least square means between intensities with corresponding 95% confidence intervals. Correlations were interpreted as follows: weak = 0.2–0.5; moderate = 0.5–0.8; strong: ≥ 0.8.

## Results

**FIGURE 2:**
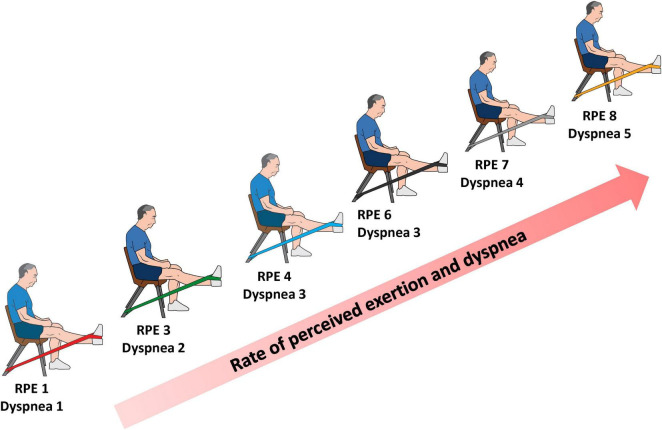
Diagram representation of rate of perceived of exertion (RPE) and dyspnea during incremental elastic resistance exercise.

**FIGURE 3:**
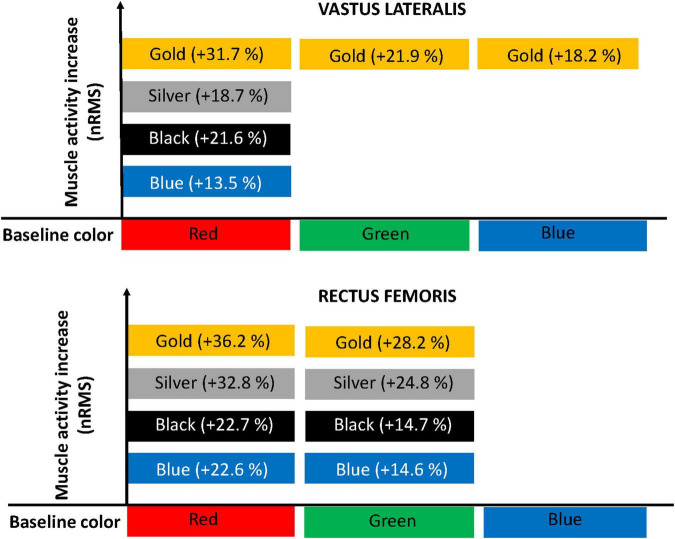
Diagram representation of increment of muscle activity. For the vastus medialis muscle, there were no root-mean-square (nRMS) increases when the resistance level progressed.

We included 14 patients (9 male), diagnosed with moderate-very severe COPD with a mean age of 67.9 ± 4.3 y, with a FEV_1_ of 47.3 ± 19.3% of predicted value. Table 1 shows the demographic and clinical data of the participants. Table 2 and Figure 2 show the acute symptoms of the participants during the different resistance levels. Table 3 shows the nRMS least squares means of the different elastic resistances. In general, the red color reported the lowest numerical mean nRMS values, while the gold color reported the highest. Table 4 and Figure 3 show the differences of least squares means between the different elastic resistances. For the VL, a nRMS increase was evident from a two-level increment when using the red color. When using the green color, a four-level increase was needed to increase nRMS. When using the blue color, a three-level increase was needed to increase nRMS. There were no nRMS differences when the black, silver and golden colors were used. For the VM, there were no nRMS increases when the resistance level progressed. A non-significant result was found when a five-level increase was performed when using the red color.

**TABLE 1:** Baseline characteristics.

Variable	
**Sex**	
Male	9 (64.3%)
Female	5 (35.7%)
Age (y)	67.9 ± 4.3
Weight (Kg)	71.4 ± 13.9
Height (cm)	169 (158–171)
BMI	26.4 ± 5.6
**GOLD classification** I II III IV	0 (0%) 5 (35.7%) 5 (35.7%) 4 (28.6%)
FEV_1_ (L)	1.34 ± 0.57
FEV_1_ (% of predicted)	47.3 ± 19.3
**mMRC** 0 1 2 3 4	1 (7.1%) 3 (21.4%) 6 (42.9%) 2 (14.3%) 2 (14.3%)
6MWD (m)	403 ± 110
CAT	22 (15–27)
SGRQ	43 (33–62)
Charlson index	4 (3–5)
Oxygen saturation (%)	95.6 ± 1.8
Hear rate (bpm)	80.0 ± 17.7

6MWD, six-minute walk distance; BMI, body mass index; CAT, COPD assessment test; FEV1, forced expiratory volume during the first second; GOLD, global initiative for chronic obstructive lung disease; mMRC, modified medical research council; SGRQ, Saint George respiratory questionnaire. The values are expressed in mean ± standard deviation or median (P25–P75).

**TABLE 2:** Acute symptoms during the different resistance levels.

Resistance level	Rate of perceived exertion		Dyspnea		Quadriceps fatigue		Oxygen saturation		Heart rate	
	Mean	SD	Mean	SD	Mean	SD	Mean	SD	Mean	SD
Red	1.3	1.2	1.3	1.7	1.0	1.5	95.1	2.4	80.2	17.9
Green	3.2	1.8	1.8	1.9	2.4	2.4	95.2	2.2	81.1	17.6
Blue	4.1	1.7	2.6	2.3	3.4	2.6	95.1	2.8	81.2	18.1
Black	5.6	1.9	3.4	3.0	5.3	2.4	95.6	2.1	81.7	18.8
Silver	6.7	1.7	3.5	3.2	6.1	2.7	95.5	2.1	84.7	19.7
Gold	7.9	1.7	4.7	3.3	6.2	3.1	95.4	2.1	83.9	20.6

**TABLE 3:** Normalized root mean square least squares means of the different resistance levels.

Muscle	Resistance level	Mean nRMS	Standard error	Lower	Upper
Vastus lateralis	Red	80.3	6.6	67.0	93.6
	Green	90.1	6.6	76.8	103.4
	Blue	93.8	6.6	80.5	107.1
	Black	101.9	6.6	88.6	115.3
	Silver	99.0	7.1	84.7	113.3
	Gold	112.0	7.6	96.7	127.2
Vastus medialis	Red	92.2	8.1	76.1	108.4
	Green	93.6	8.1	77.4	109.7
	Blue	104.5	8.1	88.3	120.6
	Black	107.0	8.1	90.8	123.1
	Silver	110.1	8.7	92.6	127.6
	Gold	108.5	9.4	89.7	127.3
Rectus femoris	Red	61.5	6.5	48.6	74.5
	Green	69.6	6.5	56.6	82.5
	Blue	84.1	6.5	71.2	97.1
	Black	84.2	6.5	71.3	97.2
	Silver	94.3	7.0	80.3	108.3
	Gold	97.7	7.5	82.7	112.7

nRMS, normalized root mean square.

**TABLE 4:** Differences of least squares means between the different elastic resistances.

Muscle	Vastus lateralis														
Color vs color	Red	Red	Red	Red	Red	Green	Green	Green	Green	Blue	Blue	Blue	Black	Black	Silver
	Green	Blue	Black	Silver	Gold	Blue	Black	Silver	Gold	Black	Silver	Gold	Silver	Gold	Gold
Estimate	−9.8	−13.5	−21.6	−18.7	−31.7	−3.7	−11.8	−8.9	−21.9	−8.2	−5.2	−18.2	3.0	−10.0	−13.0
standard error	6.4	6.4	6.4	6.9	7.4	6.4	6.4	6.9	7.4	6.4	6.9	7.4	6.9	7.4	7.7
*P* value	0.132	**0.041**	**0.001**	**0.009**	**<0.0001**	0.572	0.071	0.206	**0.005**	0.210	0.456	**0.017**	0.672	0.181	0.098
Lower	−22.7	−26.4	−34.5	−32.6	−46.5	−16.5	−24.7	−22.8	−36.7	−21.0	−19.1	−33.1	−10.9	−24.9	−28.4
Upper	3.1	−0.6	−8.8	−4.8	−16.8	9.2	1.1	5.0	−7.0	4.7	8.7	−3.3	16.9	4.8	2.5

**Muscle**	**Vastus medialis**														

Color vs color	Red	Red	Red	Red	Red	Green	Green	Green	Green	Blue	Blue	Blue	Black	Black	Silver
	Green	Blue	Black	Silver	Gold	Blue	Black	Silver	Gold	Black	Silver	Gold	Silver	Gold	Gold
Estimate	−1.3	−12.2	−14.7	−17.9	−16.3	−10.9	−13.4	−16.6	−14.9	−2.5	−5.6	−4.0	−3.2	−1.5	1.6
Standard error	8.3	8.3	8.3	9.0	9.6	8.3	8.3	9.0	9.6	8.3	9.0	9.6	9.0	9.6	10.0
*P* value	0.873	0.147	0.082	0.051	0.095	0.195	0.113	0.071	0.125	0.767	0.532	0.676	0.726	0.872	0.872
Lower	−18.0	−28.9	−31.4	−35.9	−35.5	−27.6	−30.1	−34.5	−34.2	−19.2	−23.6	−23.2	−21.1	−20.8	−18.4
Upper	15.3	4.4	1.9	0.1	2.9	5.8	3.3	1.4	4.3	14.2	12.3	15.2	14.8	17.7	21.6

**Muscle**	**Rectus femoris**														

Color vs color	Red	Red	Red	Red	Red	Green	Green	Green	Green	Blue	Blue	Blue	Black	Black	Silver
	Green	Blue	Black	Silver	Gold	Blue	Black	Silver	Gold	Black	Silver	Gold	Silver	Gold	Gold
Estimate	−8.0	−22.6	−22.7	−32.8	−36.2	−14.6	−14.7	−24.8	−28.2	−0.1	−10.2	−13.6	−10.1	−13.5	−3.4
Standard error	6.6	6.6	6.6	7.1	7.6	6.6	6.6	7.1	7.6	6.6	7.1	7.6	7.1	7.6	7.9
*P* value	0.230	**0.001**	**0.001**	**<0.0001**	**<0.0001**	**0.032**	**0.030**	**0.001**	**0.001**	0.989	0.159	0.080	0.163	0.082	0.670
Lower	−21.3	−35.9	−36.0	−47.1	−51.5	−27.8	−27.9	−39.1	−43.4	−13.3	−24.5	−28.9	−24.4	−28.8	−19.3
Upper	5.2	−9.4	−9.5	−18.5	−20.9	−1.3	−1.4	−10.5	−12.9	13.2	4.1	1.7	4.2	1.8	12.5

Bold color denotes a statistically significant difference.

For the RF, a nRMS increase was evident from a two-level increment when using the red color and after a one-level increment when using the green color. There were no nRMS differences when the blue, black, silver, and golden colors were used. The association between the resistance level and each of the physiological outcome variables are shown in Table 5.

**TABLE 5:** Association between outcome variables.

Pearson correlation	RF nRMS	VL nRMS	VM nRMS	RPE	Dyspnea	Quadriceps fatigue
Resistance level (1 = red, 6 = gold)	0.46 (*p* < 0.01)	0.34 (*p* < 0.01)	0.21 (*p* = 0.06)	0.79 (*p* < 0.01)	0.40 (*p* < 0.01)	0.62 (*p* < 0.01)
The relative resistance%	0.39 (*p* < 0.01)	0.30 (*p* < 0.01)	0.19 (*p* = 0.10)	0.72 (*p* < 0.01)	0.45 (*p* < 0.01)	0.57 (*p* < 0.01)
RPE	0.54 (*p* < 0.01)	0.51 (*p* < 0.01)	0.33 (*p* < 0.01)	1	0.70 (*p* < 0.01)	0.84 (*p* < 0.01)
Dyspnea	0.47 (*p* < 0.01)	0.48 (*p* < 0.01)	0.32 (*p* < 0.01)	0.70 (*p* < 0.01)	1	0.66 (*p* < 0.01)
Quadriceps fatigue	0.52 (*p* < 0.01)	0.50 (*p* < 0.01)	0.37 (*p* < 0.01)	0.84 (*p* < 0.01)	0.66 (*p* < 0.01)	1

The relative resistance was normalized to the maximum resistance level achieved. RPE, rate of perceived of exertion.

## Discussion

The main finding was that heavy elastic resistance exercise seems to be feasible in COPD participants, without causing high dyspnea increments and with a stable cardiorespiratory response (heart rate and dyspnea). In most of the cases, at least a two-level resistance increase was needed to obtain a significant nRMS increase during knee extensions in COPD participants. In fact, a real nRMS increase was only evident at the VL and RF, revealing that in spite of increasing the elastic resistance, the VM was not further stimulated. To our knowledge, this is the first study demonstrating specific nRMS values for different quadriceps muscles during a typical rehabilitative exercise with progressive resistance, which may help to individualize exercise dosing.

The nRMS changes were mostly observed during low-moderate resistance levels, while during the three heaviest elastic resistances (black, silver, and gold colors), there were no changes, in contrast with a similar previous study in participants with hemophilia (26). In fact, the greatest between-resistance mean differences in our study were found during the first resistance level. This might be explained by a greater opportunity window for increasing nRMS during the lower resistances and lower accumulated neuromuscular fatigue. In addition, intrinsic muscle changes in COPD participants due to early metabolite accumulation (27) and reduced fiber conduction velocity (28) while exercising may have caused early fatigue and reduced the possibility of increasing nRMS after this point. It is worth mentioning that some differences across the quadriceps muscles were noted. For instance, it seems that the VL needs a greater resistance increment than the RF to increase its nRMS. The VL increased its nRMS during the knee extension with the blue color (fourth heaviest resistance) while nRMS at the RF was not increased further after the green color (fifth heaviest resistance). In line with our findings, a recent similar study conducted among participants with severe hemophilia reported that increasing 2–3 elastic resistance levels was needed to increase nRMS in the quadriceps muscle (26).

Another between-muscle difference was that VL and VM had greater general nRMS than RF. Comparable results have been found in participants with COPD during an isometric knee extension (29) or in healthy adults during dynamic knee extensions (30). This greater muscle activation at least at the VL could be partially explained by a stronger force-generating capacity (31). Specifically, it was estimated that the VL and VM had a contribution of 40 and 25% of the quadriceps strength, respectively, while the RF and vastus intermedius contributed 35% (31). This, in conjunction with the more linear EMG/force relationship at the VL compared with the RF and VM might explain why this muscle was the only one showing some differences between the heaviest elastic resistance levels (32). In addition, a shift from fiber type I to II in the VL is a typical skeletal muscle alteration among COPD participants (33). Little information exists about the differences between quadriceps muscles’ EMG activity during exercises in COPD (28, 34). In healthy individuals, a difference in histochemical and EMG characteristics between VL and VM has been reported (35, 36). Our study found the same trend, where the absence of nRMS changes in the VM despite the progressive resistance provided was observed. In COPD participants using a multichannel surface EMG, it has been reported that fatigue indexes correlate better with lung function and exercise tolerance for vastus medialis than for vastus lateralis (34). This is likely due to the altered neuromuscular response in COPD participants and some of the dissimilarities mentioned above between quadriceps muscles (28, 34–36). The latter suggests a decreased ability to activate the quadriceps maximally in COPD participants, particularly in VM, probably dependent on altered conduction velocity, motor unit recruitment, and firing rate (28, 34, 37). However, future studies are needed to prove this assumption.

Our correlations between the color of the band or the relative resistance and VM nRMS were not significant, but were significant for VL nRMS and RF nRMS. Likewise, VL nRMS and RF nRMS were more correlated with RPE, dyspnea, and quadriceps fatigue than VM nRMS (see Table 5). This might have relevant clinical implications, especially for those with more advanced disease and less exercise tolerance. For example, in this specific case and muscle, a lower resistance could be used to provide similar nRMS but with a lower RPE, dyspnea and quadriceps fatigue, which may facilitate training and adherence. Dyspnea or muscle fatigue usually limit or stop exercise practice among COPD participants before the skeletal muscles are maximally stressed (17). In our case, dyspnea, quadriceps fatigue and RPE increased in a dose-response fashion while progressing resistance. However, dyspnea was maintained within commonly used and recommended ranges of breathlessness intensity for people with COPD (20). Furthermore, we found that quadriceps fatigue and especially RPE were more correlated with the resistance progressions than dyspnea, which did not increase to the same extent, suggesting that these variables can especially limit resistance training and should be monitored during resistance training for pulmonary rehabilitation. Based on our results, RPE was the acute symptom with the greatest and more consistent increase likely due to its moderate association with RF and VL nRMS and dyspnea, and especially due to its strong correlation with muscle fatigue. Notably, a study among COPD participants reported no cardiorespiratory changes during knee extensions at low and high intensities, but RPE did increase with the latter (38). In accordance, we found that heart rate and oxygen saturation were stable during the different resistance levels, whereas the other acute symptoms, and especially RPE, increased.

It must be taken into account that our results cannot be extrapolated to other exercises or exercise variations (e.g., performing knee extensions with both limbs at a time). In addition, since mostly male participants were included, generalization for females should be interpreted with caution. It also needs to be considered that all the measurements were conducted during three repetitions per condition, which may differ when targeting higher volumes or different muscles. However, this approach is appropriate and is usually used in literature to evaluate nRMS during multiple resistances/intensities to avoid accumulated fatigue (24, 26, 39), which can be especially relevant in COPD participants (28). Lastly, since resistance levels were not randomized, this may have particularly impacted symptom reporting if participants expect to be more symptomatic as the resistance increase.

In conclusion, heavy elastic resistance exercise seems to be feasible in COPD participants, without causing high dyspnea increments and with a stable cardiorespiratory response. In general, COPD participants need to increase at least two elastic resistance levels to obtain a real RF and VL nRMS increase during knee extensions, while there is no nRMS progression for the VM. There is no nRMS progression between the three heaviest elastic resistances, although these levels could be used to increase muscle fatigue or RPE. Dyspnea, quadriceps fatigue, and especially RPE increase in a dose-response fashion and are correlated with the relative resistance, the resistance level and nRMS. These results may help to individualize exercise dosing during elastic resistance training in participants with COPD.

## Data availability statement

The raw data supporting the conclusions of this article will be made available by the authors, without undue reservation.

## Ethics statement

The studies involving human participants were reviewed and approved by Northern Metropolitan Health Service of Santiago, Chile. The patients/participants provided their written informed consent to participate in this study.

## Author contributions

JC, BG-G, LA, and CC-M conceived and designed study. JC, BG-G, CC-M, ÁO-V, and NS-C collected raw data. BG-G, LA, and CC-M completed all data analyses. JC and RT-C wrote the manuscript. All authors discussed the results and contributed to the final manuscript.

## Footnotes

1
http://www.seniam.org/

## Abbreviations

COPD: chronic obstructive pulmonary disease
nRMS: normalized root mean square
RF: rectus femoris
VL: vastus lateralis
VM: vastus medialis
RPE: rate of perceived exertion
sEMG: surface electromyography.
